# Senior Secondary School Food Literacy Education: Importance, Challenges, and Ways of Improving

**DOI:** 10.3390/nu10091316

**Published:** 2018-09-17

**Authors:** Janandani Nanayakkara, Claire Margerison, Anthony Worsley

**Affiliations:** Institute for Physical Activity and Nutrition, School of Exercise and Nutrition Sciences, Deakin University, Geelong 3220, Australia; claire.margerison@deakin.edu.au (C.M.); tonyw@deakin.edu.au (A.W.)

**Keywords:** food literacy education, senior secondary school, stakeholders, adolescents

## Abstract

Food literacy education at senior secondary school can provide both immediate and long-term benefits for adolescents. The exploration of multiple stakeholder groups’ opinions regarding the importance, roles, and challenges of school food literacy education, and their suggestions for its improvement, will help the design and execution of future food literacy-related curricula and programmes. This study explored a broad range of Australian and international food, health, and education professionals’ opinions regarding senior secondary school food literacy education through an online survey. One hundred and fifty-five food, health, and education professionals completed this survey between April and October 2017. Overall, the respondents strongly supported the need for food literacy education for senior secondary school students. Their suggestions for improving this form of education included: incorporation of relevant and up-to-date content, the presence of strong practical components, offering food literacy as compulsory subjects or the incorporation of food literacy concepts into compulsory core subjects. Moreover, they proposed the active contribution of both internal and external stakeholders in the planning and delivery of this education to upgrade its quality and relevance. Overall, the findings suggest that a wide range of food, health, and education professionals are highly supportive of senior secondary school food literacy education and their constructive suggestions should be considered in school food literacy education enhancement efforts. Education authorities should seek ways of involving different stakeholders, including food-related professionals, in the design and delivery of food literacy education, and future studies should explore the best mechanisms for such involvement.

## 1. Introduction

Food literacy is an emerging concept that has been heavily influenced by health professionals, nutritionists, and home economists and fundamentally aims at achieving personal health-related goals [1,2,3,4,5]. A well-known definition of food literacy is “the scaffolding that empowers individuals, households, communities or nations to protect diet quality through change and strengthen dietary resilience over time. It is composed of a collection of interrelated knowledge, skills, and behaviours required to plan, manage, select, prepare, and eat food to meet needs and determine intake” [1] (p. 54). However, recently some broader definitions of food literacy have emerged incorporating other aspects of food such as environmental sustainability and social equity [6,7,8]. As an example, Cullen and colleagues defined food literacy as follows: “Food literacy is the ability of an individual to understand food in a way that they develop a positive relationship with it, including food skills and practices across the lifespan in order to navigate, engage, and participate within a complex food system. It is the ability to make decisions to support the achievement of personal health and a sustainable food system considering environmental, social, economic, cultural, and political components” [8] (p. 143). Although there is no a universally accepted definition, these emerging and broader definitions suggest that food literacy includes at least four major domains as follows: (i) the food system from production to waste, (ii) the effect of food on health and wellbeing, (iii) the wider context of the food system including social, economic, cultural, environmental, and political factors, and (iv) the development of skills and behaviours related to food [1,6,8,9,10].

In many educational contexts around the world, some components of food literacy such as food and nutrition knowledge and food planning and preparation skills, have been taught to adolescents through home economics and other related subjects for over 100 years [11,12]. In recent years, there has been increased interest in improving school food and nutrition education and the raising of students’ food-related knowledge and cooking skills. This is partly due to the increase in the prevalence of diet-related diseases (i.e., diabetes, heart diseases, and obesity) and the recognition of the role of poor dietary patterns and lack of food preparation skills in the etiology of these adverse health conditions [12,13]. On the bright side, new school food literacy-related programmes and curricula that encompass both individual-health goals and broader environmental, social, political, and economic aspects of food literacy are flourishing in certain countries. For instance, in 2017, the Victorian Curriculum and Assessment Authority in Australia introduced a new elective curriculum named ‘Victorian Certificate of Education Food Studies (VCE Food Studies)’ for senior secondary school students in Victoria, Australia [14]. Food preparation and nutrition are only two aspects of this curriculum; it comprises broader food literacy concepts such as food history, primary food production, food systems, environmental sustainability, and social equity in food distribution and consumption, etc. [14]. 

Schools have been identified as ‘social complex adaptive systems’ and consist of diverse agents [15]. Thus, exploration of different stakeholders’ opinions regarding the importance of school food literacy education, the challenges associated with this form of education, and ways of overcoming these barriers will provide useful insights for these agents (i.e., agencies and personnel) involved in school food literacy curricula and programme development. The authors designed a preliminary model of stakeholder involvement in secondary school food literacy education (Figure S1) that is based on both Australian and overseas literature related to different groups and sectors interested in school food, nutrition, health education, and associated policies [1,3,13,16,17,18,19,20,21,22,23,24,25,26,27,28,29,30,31,32,33,34,35,36,37]. It shows that school food literacy education involves many different stakeholders including school personnel, students and their immediate social networks, food system professionals, government agencies, non-government organizations, private sector industries, and media. 

Previous overseas and Australian researchers have explored some of these stakeholders’ opinions regarding various aspects of school food literacy education [13,22,28,29,30,33,38]. These studies suggest there is an important research gap in regard to the exploration of different stakeholder groups’ opinions regarding school food literacy education. That is, the under-examination of food system professionals’ and teachers’ opinions regarding the senior secondary school food literacy education (i.e., last years of schooling) and of the previously mentioned new VCE Food Studies curriculum in Australia. Food system professionals have knowledge and skills in various food-related areas and they are aware of students’ career prospects in food-related areas [24,39]. This makes them an important group who can provide valuable insights into food-related school curricula and programme development. In addition, exploration of teachers’ opinions regarding food literacy education is important to identify their resource needs and the challenges they face in curriculum delivery. Accordingly, the authors explored food system professionals’ and teachers’ opinions of these two topics using qualitative studies and the results have been reported previously [40,41,42].

The findings of these qualitative studies [40,41,42] were used to design a quantitative survey of food professionals’ and teachers’ opinions of these two areas. The specific objectives of this study were to explore these professionals’: (i) opinions of the importance of food literacy education for senior secondary school students, (ii) support for the inclusion of different food-related topics in the senior secondary school food literacy curriculum, (iii) views of the importance of students’ food literacy-related activities and assessment tasks, (iv) perceptions of barriers and challenges for delivering food literacy education to senior secondary school students, and (v) suggestions for improving the quality and relevance of senior secondary school food literacy education. 

## 2. Materials and Methods 

### 2.1. Survey Instrument and Administration of Survey

Themes identified in the two previous qualitative studies conducted by the authors [40,41,42] informed the specific areas to be explored in this study and, accordingly, the questionnaire included six main sections. Thirty-one statements (from two previous qualitative studies) related to the importance of food literacy education for senior secondary school students, students’ activities and assessment-related tasks in food literacy education, and the challenges associated with food literacy education were incorporated into sections A, C, and D of the questionnaire, respectively. An exploratory sequential mixed methods study design was employed [43,44]. The major topics of the new VCE Food Studies curriculum were incorporated into section B of the questionnaire. The main sections and the associated sub-sections of the questionnaire are shown below.

#### 2.1.1. Section A: The Importance of Food Literacy Education for Senior Secondary School Students

Respondents were asked to rate their agreement with 10 statements related to the importance of food literacy for senior secondary school students. Five category rating scales were used (strongly agree (coded as 1), agree (coded as 2), no idea/ not sure (coded as 3), disagree (coded as 4), strongly disagree (coded as 5)). After inspection of the data distribution, it was decided to aggregate these categories into ‘agree’ (codes 1 and 2) and ‘disagree’ (codes 3, 4, and 5) to facilitate the interpretation of cross-tabulation analyses.

#### 2.1.2. Section B: The Inclusion of Different Topics in Senior Secondary School Food Literacy Education 

Respondents were asked to rate their agreement with the inclusion of different food-related topics in the food literacy curriculum for senior secondary school students. A list of 35 topics was presented under four sections. The five category rating scales were employed (as above) and similar category aggregation was used to facilitate the interpretation of cross-tabulation analyses. Cronbach’s alpha for the items in the four sections were: 0.68 (history of food), 0.82 (the food system), 0.82 (the science of food and influences on food consumption), and 0.87 (food system-related issues and challenges), indicating acceptable internal consistency reliability [45].

#### 2.1.3. Section C: Students’ Activities and Assessment-Related Tasks in Food Literacy Education

The respondents were asked to rate their agreement with seven statements about students’ activities and assessment-related tasks. Again, five category rating scales were employed (as above) and similar category aggregation was used to facilitate the interpretation of cross-tabulation analyses.

#### 2.1.4. Section D: Barriers and Challenges for Food Literacy Education for Senior Secondary School Students

The respondents rated their agreement with 14 statements related to barriers and challenges for food literacy education for senior secondary school students. The same scale categories and aggregations were used as in the previous sections. Then, the respondents were asked ‘Are there any other barriers for food literacy education for senior secondary school students?’ Space was provided for them to record their answers verbatim. 

#### 2.1.5. Section E: Improving the Quality and Relevance of School Food Literacy Education

The respondents were asked ‘Please suggest how the quality and relevance of school food literacy education can be improved’ to get a better understanding of food, health, and education professionals’ opinions of school food literacy education and to confirm their ratings of different aspects of this form of education (i.e., convergent mixed methods study design) [43,44]. Again, the respondents recorded their views in a space that was provided.

#### 2.1.6. Section F: Professional and Demographic Characteristics

The professional and demographic characteristics of the respondents were obtained through questions about the following areas:

- Post-school qualification(s) (nine options: education, health, physical education, hospitality management, science, agriculture, nutrition, food science, other);
- Area(s) of food-related experience (14 options: education, agriculture or horticulture, food manufacturing, food distribution, retailing, food marketing, food service, health/nutrition promotion, dietetics, environmental agencies or groups, communications, university/research, government regulatory agency, other);
- Years worked/involved in food-related area(s) (a continuous variable, and then coded as 1 = 1–10 years, 2 = 11–20 years, 3 ≥ 20 years);
- Current roles(s) (nine options: educator/teacher, health professional/service provider, researcher, manager/administrator, technologist, practitioner, dietitian, business owner, other; then coded as 1 = food industry professionals (FIP), 2 = health professionals (HP), 3 = school teachers (TH));
- Age (a continuous variable, then coded as 1 ≤ 40 years, 2 ≥ 40 years);
- Gender (four options: male, female, other, prefer not to say);
- Current residence (two options: Australia, other countries).

The content validity of the survey instrument was determined through review of the instrument by three food-related professionals who had experience in mixed-method research [45]. The questionnaire’s structure, flow, and wording were modified based on the experts’ suggestions. The modified version was pre-tested on four food and nutrition professionals. Based on their suggestions, a few minor changes were made to make the questionnaire more user-friendly and this version was uploaded to the Qualtrics online platform (http://deakinhealth.qualtrics.com). The second author completed the online survey as a trial and suggested a few changes to the wording and flow of questions, and these suggestions were incorporated into the final survey questionnaire. A link to the plain language statement was included in the survey. An anonymous link to the final version of the questionnaire was used for the data collection. The survey was conducted in April–October 2017. Ethics approval for this study was obtained from Deakin University Health Faculty Ethics Advisory group (HEAG-H 15_2017).

### 2.2. Design and Sampling

Three strategies were employed to recruit respondents for this survey. 

Strategy 1: Twenty-five organisations that deal with food-related areas (i.e., food policy, food security, health and well-being, food regulation, environmental sustainability, home economics, etc.) in Australia and overseas were identified through discussions between the authors. The heads, or suitable alternative ranking personal, of these organisations were approached and requested to advertise or distribute the survey invitation flyer among their members. 

Strategy 2: Food and nutrition-related professionals (approximately 85 professionals) attached to universities and other professional organisations in Australia and overseas were identified through a thorough web search, and their email addresses were obtained through publicly-available web sites. Furthermore, email addresses of a group of food technologists and home economics professionals were obtained from two conference participants lists. An email invitation, along with the survey link, was sent to these professionals.

Strategy 3: Social media platforms (Facebook, LinkedIn, and Twitter) were employed to increase the reach of the survey among potential participants. Australian and overseas Facebook groups related to food, nutrition, health, gardening and farming, agriculture and horticulture, hospitality, and food education professionals and teachers were identified. Ten different Facebook advertisements containing the survey invitation and link were designed to attract the different target groups and then these adverts were shared among the previously identified Facebook groups. In addition, three rounds of paid Facebook advertisements were employed (that is, the previously mentioned adverts were boosted). 

The preliminary analysis of the respondents’ demographic profile revealed that the sample lacks marketing professionals. Therefore, in collaboration with a senior professional in marketing education, a group of junior and senior food and beverage marketing managers (in retail and wholesale marketing establishments) in Australia was identified. A short message containing the link to the survey was sent to these identified professionals via the LinkedIn platform. The survey invitation was also sent to a senior home economics professional in Australia who was asked to distribute the survey to her professional network. This professional forwarded the survey invitation to both Australian and overseas home economics professionals and teachers using Twitter. 

Except for strategy 2 and the LinkedIn invitations, the authors did not send the survey invitations directly to the potential professionals (i.e., invitations were sent via relevant organizations or invitations were posted in social media sites). Therefore, the total number of professionals who received the survey invitation was unable to be obtained by the authors.

### 2.3. Data Analysis

Quantitative analyses: The responses to the closed answer questions were analysed using SPSS statistical software (Version 24, 2016, IBM Corporation, Armonk, NY, USA). Cross tabulation (Chi-square) analyses were performed to examine bivariate associations between several categorical variables (current professional role (FIP, HP, and TH), experience of food-related areas (1–10 years, 11–20 years, and >20 years), age (<40 years and ≥40 years), gender (male or female), type of educational qualification (education, health, physical education, hospitality, science, agriculture, nutrition, and food science), residence (Australia or overseas)), and the respondents’ opinions of different aspects of senior secondary school food literacy education (role of and need for food literacy education, curriculum contents, students’ activities and assessment, and barriers for food literacy education). In view of the use of multiple significance tests, a *p* value of less than 0.01 was selected as the level of significance, to guard against type 1 error [46].

Qualitative analyses: One hundred professionals provided 155 written responses to the question ‘Please suggest how the quality and relevance of school food literacy education can be improved’. These responses were extracted from the Qualtrics online platform and uploaded to NVivo (Version 11, 2015, QSR International Pty Ltd., Doncaster, Victoria, Australia), and analysed using the template analysis technique [47,48]. The first author developed an initial template, comprised of ‘a priori’ codes (themes identified after reading the first 30 responses) and data were coded using the template [47,48]. The authors met regularly during the data coding process and discussed the findings. New themes and subthemes were developed during the remaining data coding process as required. The final template comprising themes and subthemes is described in the Results section below. Verbatim quotes are used to illustrate the major findings. Along with NVivo coding, the professionals’ responses to the above question were loaded, as Word files, into the Leximancer thematic analysis program (Version 4, 2011, Leximancer Pty Ltd., St Lucia, Queensland, Australia) and a concept map was generated. Leximancer is qualitative data analysis software that generates themes and related concepts automatically from qualitative data. The themes created from manual coding using NVivo were compared with the concept map generated by the Leximancer software (Figure S2). After inspecting both sets of results, the authors concluded that there was good agreement between two sets of themes, confirming the reliability of the results generated through manual coding.

## 3. Results

### 3.1. Respondents’ Characteristics

One hundred and fifty-five food, health, and education professionals completed the survey out of the 282 professionals who commenced the survey, giving a completion rate of 55% (the survey response rate could not be calculated, as the number of respondents who received the survey invitation was not able to be obtained). The demographic and professional backgrounds of the respondents are shown below in Table 1. The respondents were predominantly females and just over half (55%) were less than 40 years old (Table 1). The majority of the respondents were from Australia (69%) and the remaining 31% were from 14 other countries (New Zealand, Canada, Colombia, Denmark, England, India, Ireland, Norway, Pakistan, Singapore, South Korea, Sri Lanka, the USA and Vietnam). Nearly all of the professionals had post-secondary school education qualifications and the most common area of qualification was nutrition, followed by education and food science. Nearly half of the respondents (45%) were health professionals (HP). They had varying levels of experience in food-related areas (Table 1).

### 3.2. Respondents’ Views of the Importance of Food Literacy Education for Senior Secondary School Students 

Nearly all the respondents (99%) agreed that ‘There should be continuity between primary and secondary school food literacy education’. More than 90% (92–94%) agreed with the need for, and roles of, senior secondary school food literacy education (Table 2). Over three-quarters (78%) agreed that ‘Food literacy should be a compulsory subject for senior secondary school students’ and nearly two-thirds (63%) agreed that ‘Food literacy should be offered as a separate and individual subject’. Only one-third of respondents (34%) agreed that ‘It is difficult to include food literacy concepts in other senior secondary school subjects’ (Table 2).

### 3.3. Respondents’ Views about the Inclusion of Different Topics in Senior Secondary School Food Literacy Education 

The majority of professionals agreed with the inclusion of ‘causes and prevention of food wastage’ (98%), followed by ‘planning and preparation of food’ and ‘safe food handling’ (97%) topics in the senior secondary school food literacy curriculum. Between 90–95% of the respondents agreed with the inclusion of 15 out of the remaining 32 topics (Table S1). Between 80–90% of the respondents agreed with the inclusion of 11 other topics (Table S1). The topics that attracted the least support for inclusion were ‘hunter gatherer and early agricultural food systems’ and ‘transferring domestic food skills to small scale commercial settings’ (both 66%) (Table S1).

### 3.4. Respondents’ Views of Students’ Activities and Assessment-Related Tasks

The majority of the respondents (>90%) agreed with the importance of practical lessons in senior secondary school food literacy education, including cooking based practicals and excursions (Table S2). More than 80% agreed with the importance of guest lectures (88%) and short-term internships and industry placements (81%) (Table S2). Only 40% agreed with the statement ‘Development of design briefs (new food product development plans) does not help to develop food literacy skills’ (Table S2).

### 3.5. Respondents’ Views of the Barriers and Challenges Facing Food Literacy Education for Senior Secondary School Students 

More than 80% of respondents agreed with ‘competition with other subjects’, ‘exposure of students to conflicting food and nutrition-related information through different media’, and ‘perceived low academic status of subject by school managements, parents, and students’ as barriers facing senior secondary school food literacy education (Table S3). Lower, but still substantial, proportions of the respondents (65–78%) agreed with seven other statements related to barriers (Table S3). The lowest agreement (both 50%) was regarding the following barriers: ‘lack of resource sharing among teachers’, and ‘lack of students’ interest on holistic food system approach in food literacy education’ (Table S3). 

Thirty-two respondents answered the question: ‘Are there any other barriers for food literacy education for senior secondary school students?’ The most common barriers they mentioned included ‘low awareness among students and general society about the importance of food literacy education’, ‘inadequate support from policy makers to improve food literacy education’, and ‘inadequacy of food literacy subjects in junior school years’.

### 3.6. Differences in Respondents’ Views of the Different Aspects of Food Literacy Education Based on Their Demographic and Professional Characteristics

There were a few differences between various categories of professionals. However, the prominent differences were as follows:

- More school teachers (55%) agreed that ‘It is difficult to include food literacy concepts in other senior secondary school subjects’ compared to food industry professionals (28%) and health professionals (21%) (Chi-sq = 15.514, *p* < 0.001).
- Fewer food industry professionals (53%) agreed with the inclusion of ‘Indigenous food practices’ in senior secondary school food literacy curriculum compared to health professionals (84%) and teachers (94%) (Chi-sq = 22.857, *p* < 0.001). Similarly, fewer men (56%) agreed with the inclusion of ‘Indigenous food practices’ compared to women (86%) (Chi-sq = 13.498, *p* < 0.001).

### 3.7. Suggestions for Improving the Quality and Relevance of School Food Literacy Education

Thematic analysis of the respondents’ answers to the question ‘Please suggest how the quality and relevance of school food literacy education can be improved’ resulted in three major themes. They were:

1. Changes to the curriculum contents and delivery,
2. Changes in school setting, and
3. Collaboration of and support from external settings.

#### 3.7.1. Theme 1: Changes to Curriculum Content and Delivery

Improving the status of food literacy subjects: Twenty-two respondents suggested that food literacy subjects need to be given proper value and recognition among other senior secondary school subjects. Seven of them suggested that offering food literacy subjects as compulsory and core subjects for senior secondary school students would enhance the status and value of food literacy subjects.

“Food literacy education tends to only be available in elective subjects for senior secondary students. Including this in core subjects may be more beneficial.”

(ID 68/Health professional)

Five respondents commented that food literacy education should be started in primary school and continue throughout secondary school.

Incorporate content that is relevant and up-to-date: Nineteen respondents mentioned that food literacy subjects should be framed in a way that provides knowledge and skills that are relevant and useful for the students. The knowledge and skills that they mentioned included: food planning and preparation, understanding the role of food in health, food trends, and present and upcoming food-related problems. For instance:

“Educational outcomes must include practical food skills to enable students to plan, shop and cook nutritious balanced meals for the health of themselves and their families, as well as the critical thinking skills to analyse food and nutrition myths, and misleading marketing.”

(ID 40/Health professional)

Moreover, they mentioned that the subject content needs be updated regularly and should be based on current, emerging, and evidence-based information.

“Improving the status of the subject but showing how food is based on the sciences and that it is woven into every aspect of our lives and can be used to deliver all subjects in education.”

(ID 119/Teacher)

Have a strong practical component: Ten respondents emphasised that food literacy subjects should have a strong practical component that enables students to develop their food-related skills.

“A combination of classroom and practical activities that the students can be involved in to stimulate interest and knowledge on food literacy.”

(ID 114/Food Industry professional)

#### 3.7.2. Theme 2: Changes in School Settings

Internal stakeholders’ involvement in food literacy education: Thirty-seven respondents (38 comments) suggested having active involvement and support from school management, teachers, students, and parents for improvement of quality and relevance of food literacy education. The majority of them (*n* = 26) wrote about the teachers. They believed that teachers need to have a sound understanding of food literacy concepts and present food-related issues. They suggested recruiting well-qualified professionals to the food literacy teaching profession and providing continuous professional development opportunities to acquire up-to-date food literacy knowledge and skills.

“Regular professional forums aimed to improve knowledge and skills of practising teachers.”

(ID 24/Health professional)

Seven respondents (seven comments) proposed that school management should acknowledge the importance of food literacy subjects and allocate adequate resources accordingly.

“Administration to place a significant importance in the subject.”

(ID 124/Teacher)

Four respondents mentioned that opportunities should be given to senior secondary school students to contribute to food literacy curriculum development and active involvement in school food-related activities such as school canteen menu design.

“Involve students in supporting healthier food environment at school—e.g., food waste management, healthier foods at canteens, in vending machines, kitchen gardens, food swaps etc.”

(ID 20/Health professional)

Allocation of more resources for food literacy subjects: Twenty-six respondents (27 comments) recommended that more resources should be allocated for food literacy education. The resources they mentioned included online platforms and apps with food literacy-related updated information and activities for students and teachers, more funding, adequate time allocation for food literacy subjects in school timetables, improved infrastructure, and a facilitating school environment that reinforces the facts students learn.

“The school environment needs to reflect what is taught in the classroom so needs a whole school commitment to a healthy eating environment. Food activities such as school gardens, produce fairs (incorporating cultural aspect), healthy fund raising activities can support.”

(ID 39/Health professional)

“More time for prep and time to carry out the lessons.”

(ID 119/Teacher)

#### 3.7.3. Theme 3: Collaboration and Support from External Settings

Seventeen respondents (18 comments) mentioned several ways of eliciting support from outside food professionals and organisations to improve the quality and relevance of senior secondary school food literacy education. One suggestion was the exploration and development of career pathways for students who take food literacy subjects in collaboration with food-related professionals and organisations such as farmers, nutritionists, and universities and Technical and Further Education Institutes (TAFEs).

“Partner with university programs to show the future pathways.”

(ID 147/Food industry professional)

It was also suggested that input from food-related professionals in food literacy curricula development should be sought. As an example, one respondent wrote:

“The curriculum must be informed by nutrition/food science professionals who also understand relevant food issues and nutritional needs for adolescents.”

(ID 82/Health professional)

Other suggestions included the involvement of community food movements/groups, food industry professionals, and hospitality professionals in delivering some aspects of school food literacy programmes.

“Use production classes to cook for community group. Have members of the community come in and pair up with students to cook and talk with students one on one.”

(ID 66/Teacher)

## 4. Discussion

Some important findings arise out of this study and they are discussed briefly in this section**.**

### 4.1. Improve the Status of Senior Secondary School Food Literacy Subjects

Both the quantitative and qualitative findings showed considerable support for offering food literacy-related subjects across all the school grades and offering them as compulsory subjects. For students to gain value from food literacy education, it needs to be offered from their early years of school or even earlier (i.e., in pre-school) [30,42]. Students in the lower grades can be provided with functional and interactive food literacy knowledge and skills [3,9]. Building on this, middle (years 9 and 10) and upper secondary grades (years 11 and 12) might be provided with more applied and critical food literacy knowledge and skills [3,9] such as food ethics, food regulation, and social equity in food distribution. Consistent with the previous published findings of the authors [42,49] and a recent Australian study that explored teachers’ opinions of school food literacy education [29], the respondents indicated that making the food literacy subjects compulsory would help to raise the status and recognition of these subjects in schools. 

### 4.2. Integration of Food Literacy Concepts into Co-Subjects

Food literacy concepts can be successfully integrated into compulsory subjects such as science and maths [50,51,52] and this might be a good way to disseminate food concepts to a wide range of students and to explore these concepts through different subject contexts (e.g., science or humanities) [53]. As such, integration could be one way of overcoming some of the challenges associated with the delivery of food literacy education such as competition with other subjects for time and resources and lack of recognition and support from school administrations [42,54]. However, the food teachers were more sceptical about the feasibility of cross-curriculum teaching than the food-industry and health professionals, possibly because of lack of familiarity or personal experience of problems associated with this form of teaching. Future studies should examine the broad range of stakeholders’ opinions about the integration of food literacy concepts into compulsory school subjects over offering it as a compulsory individual subject.

### 4.3. Structure of Senior Secondary School Food Literacy Curriculum 

There was widespread support for the inclusion of most of the listed topics in the senior secondary school food literacy curriculum. However, there was less enthusiasm for history-related topics, despite their importance in understanding the transition stages of the food system [53,55]. As in our previous studies [41,42], most respondents supported the integration of practical and theoretical learning. Cooking was seen as ‘an integral part of a food literacy subject’ along with gardening, visits to different food-related sites, and guest lectures. These activities would help students understand different food-related industries/areas and enhance their interest in learning about food [12,20,30,33,38].

### 4.4. Barriers Facing Senior Secondary School Food Literacy Education and Ways of Overcoming Them

Curriculum overload (or competition with other subjects) is a major challenge faced by food literacy education [21,27,29]. As mentioned earlier, this might be reduced by making food literacy subjects compulsory or integrating food literacy concepts into other subjects. However, as noted by the respondents, these solutions would require the provision of more resources (i.e., more funding, timetable time, and infrastructure facilities). 

Another challenge associated with food literacy education is the exposure of students to conflicting food and nutrition information through different media. Previous studies have also found that exposure of adolescents to food marketing via different media is associated with unhealthy eating patterns such as increased snacking and junk food consumption [56,57,58]. The senior secondary school food literacy subjects should be framed in a way that helps students to critically evaluate the facts they receive though different channels. This will enable them to distinguish reliable, evidence-based food-related information from deceiving facts and take wise decisions accordingly. As suggested by the respondents, incorporation of more up-to-date and relevant information in senior secondary school food literacy subjects and use of food-related experts’ inputs in food literacy curricula design and delivery would help to achieve this. This may enhance the students’ interest and comprehension of food literacy concepts and help them to become informed food citizens. 

Perceived low academic status of food literacy subjects by school management, parents, and students is another barrier to food literacy education [3,29]. As suggested by the respondents, the provision of opportunities for these groups to be actively involved in school food–related programmes (e.g., students being able to provide input into food literacy curriculum design and other school food-related activities) would help to develop positive attitudes towards these subjects, and consequently acknowledge the importance of school food literacy education. 

Teachers play an important role in the successful delivery of food literacy education to students. They should be passionate about food literacy education and they should have a sound understanding of food literacy concepts and food-related issues. The provision of continuous professional development (CPD) opportunities for teachers was suggested as a way of achieving this. The previous studies of the authors [40,59] complement these suggestions. In these studies, teachers repeatedly articulated the need for high quality CPD sessions to improve their knowledge and skills related to teaching new topics in the VCE Food Studies curriculum. Accordingly, all these findings emphasise the need for frequent and ongoing professional development opportunities for teachers of food literacy subjects. These professional development programmes should be carefully crafted considering the needs of teachers and their students, and the dynamic nature of food-related issues in the world [54,60,61].

Inviting the services of various food and nutrition experts such as farmers, food technologists, nutritionists, dietitians, food activists, and career consultants (i.e., external stakeholders) as guest speakers could assist in improving the quality and relevance of senior secondary school food literacy education. Furthermore, these stakeholders should be involved in food literacy curriculum design. This would help to make food literacy-related subjects relevant to students’ personal and social lives and future careers.

### 4.5. Implications for Future Research and Practice

These findings have some implications for the development and delivery of senior secondary school food literacy curricula and programmes in Australia and elsewhere. The findings of this study suggested that the broad range of food system professionals and health professionals have sound understanding of how senior secondary school food literacy-related education can be improved. Both higher level (i.e., education authorities, education ministries or departments) and lower level (i.e., schools) food literacy education design and implementation bodies should try to involve these professionals in the design and delivery of school food literacy education. However, any involvement of these external stakeholders needs to be properly planned to avoid problems such as the inclusion of other incompatible agendas (for example, product marketing in food education programmes) and loss of control over the types of information delivered to students. The education authorities and curriculum leaders should consider the suggestions provided by the professionals in this study in future secondary school food literacy-related curricula and programme execution.

The findings show the importance of starting the food literacy education in primary school and continuing it throughout the secondary and senior secondary years. The development of a national food literacy education framework similar to the ‘British framework of skills and knowledge around food, diet, and physical activity for children and young people aged 5 to 16 years’ [62] would help to structure the food literacy education programme across all school years. Countries could design their own frameworks based on overseas frameworks in addition to considering local food systems, values, and education systems. Such frameworks would facilitate curriculum leaders to design new food literacy-related curricula, and enable teachers to design lesson plans and student assessments in the light of the core competencies for the different age groups. 

Future research should examine the opinions of different stakeholder groups, especially those of under-researched groups such as curriculum leaders, bureaucrats, media, food activists groups, etc., regarding the strengths, weaknesses, opportunities, and threats facing secondary school food literacy education in Australia (or elsewhere). These findings would identify the stances of different levels of society regarding this form of education. This could initiate a broad public discussion regarding the importance of school food literacy education and help raise its status and profile in education and the broader society.

### 4.6. Strengths and Limitations 

This study had several strengths. First, it included food, health, and education professionals from 15 different countries (Australia and 14 other countries). This provides international insights into the importance of, and challenges encountered by, senior secondary school food literacy education. Second, it explored a broad range of food, health, and education professionals’ opinions of senior secondary school food literacy education. According to the best of the authors’ knowledge, this study is unique and no previous surveys have explored international food, health, and education professionals’ opinions of the aspects of food literacy education explored in the present survey. This survey was open to both Australian and overseas professionals with the aim of obtaining international food, health, and education professionals’ perspectives of senior secondary school food literacy education. However, the majority of respondents were from Australia (69%). Thus, the findings may mainly depict Australians’ views of school food literacy education. The third strength was the inclusion of both quantitative and qualitative questions in the questionnaire (i.e., convergent mixed method study design) [43,44]. This enabled better inferences to be made about the professionals’ opinions of food literacy education [43,44,63]. Another strength was the recruitment of respondents through multiple avenues, including a web search for publicly available contact details, social media, newsletters, professionals’ networks, etc. This helped to obtain a diverse sample of food-related professionals.

One of the limitations of this study was its relatively low sample size and the relatively low survey completion rate. Out of the 282 professionals who commenced the survey, only 55% completed it. The professionals’ demanding workloads may have prevented them from completing the survey at once and consequently resulted in them forgetting to complete it later. A reminder email may have helped to increase the completion rate. However, it was not possible to send such an email, as respondents’ contact details were not known and most of the respondents were invited to complete the survey via indirect methods (as mentioned earlier). For the same reason it was not possible to determine the overall response rate of the survey (both the number commencing, and the final number invited). The sample was comprised mainly of females (81%). Therefore, caution should be applied when interpreting the results, as the overall food-related professionals’ opinions of school food literacy education might be different to those of the study respondents.

## 5. Conclusions

This study highlights the importance of senior secondary school food literacy education and provides some useful suggestions for its improvement from the viewpoint of food, health, and education professionals. These findings add to ongoing discussions of ways to strengthen school food literacy education and emphasise the value of involving these professionals in such efforts.

## Figures and Tables

**Table 1:** Respondents’ demographic and professional characteristics.

Characteristics	*n*	%
**Gender ^a^**		
Female	125	81
Male	27	18
Prefer not to say	2	1
**Age (years) ^b^**		
<40 years	82	55
≥40 years	67	45
**Country of residence**		
Australia	107	69
Overseas	48	31
**Areas of post-secondary school qualifications ^c^**		
Nutrition	73	47
Education	72	46
Food Science	64	41
Health	46	30
Science	34	22
Hospitality Management	33	21
Agriculture	8	5
Physical Education	4	3
Other	52	34
**Current role**		
Food industry professional (FIP)	32	21
Health professional (HP)	70	45
Teacher (TH)	53	34
**Experience in food-related areas (years) ^d^**		
1–10	59	40
11–20	45	30
>20	45	30

^a^*n* = 154, as one respondent did not mention the gender; ^b^ *n* = 149, as six respondents did not mention their age; **^c^** The percentages do not add up to 100%, as most respondents had qualifications in more than one area; ^d^ *n* = 149, as six respondents did not mention their experience in food-related areas.

**Table 2:** Respondents’ agreement with statements related to the importance of food literacy education for senior secondary school students (years 11 and 12).

Statements	Agree (%)	Disagree (%)
There should be continuity between primary and secondary school food literacy education.	99	1
Food literacy education helps senior secondary school students develop their food skills such as meal planning, food preparation, and cooking.	94	6
Food literacy education helps senior secondary school students establish critical thinking skills about food system-related issues.	93	7
Senior secondary schooling years are appropriate to deliver broader concepts of food literacy.	92	8
Lack of food and nutrition knowledge and cooking skills in the general population and school students demands food literacy education at secondary school.	92	8
Food literacy education helps senior secondary school students to make healthier food choices.	92	8
Food literacy should be a compulsory subject for senior secondary school students.	78	22
Food literacy education helps students to choose careers in food and nutrition-related areas.	77	23
Food literacy should be offered as a separate and individual subject for senior secondary school students.	63	37
It is difficult to include food literacy concepts in other senior secondary school subjects.	34	66

## Supplementary Materials

The following are available online at http://www.mdpi.com/2072-6643/10/9/1316/s1: Figure S1. The model of stakeholder involvement in secondary school food literacy education, Figure S2. Leximancer concept map, Table S1. Percentages of respondents who agreed with the inclusion of different topics in the food literacy curriculum for senior secondary school students (years 11 and 12), Table S2. Respondents’ agreement with seven statements related to students’ activities and assessment-related tasks in food literacy education for senior secondary school students (years 11 and 12), Table S3. Respondents’ agreement on statements related to the barriers and challenges facing food literacy education for senior secondary school students.
