# Supplementary material for: Senior Secondary School Food Literacy Education: Importance, Challenges, and Ways of Improving
Source: Nutrients. 2018 Sep 17;10(9):1316. doi: 10.3390/nu10091316 (PMC6164333; doi:10.3390/nu10091316)
Supplement: Supplementary file 1 [file nutrients-10-01316-s001.pdf]

nutrients

Supplementary materials

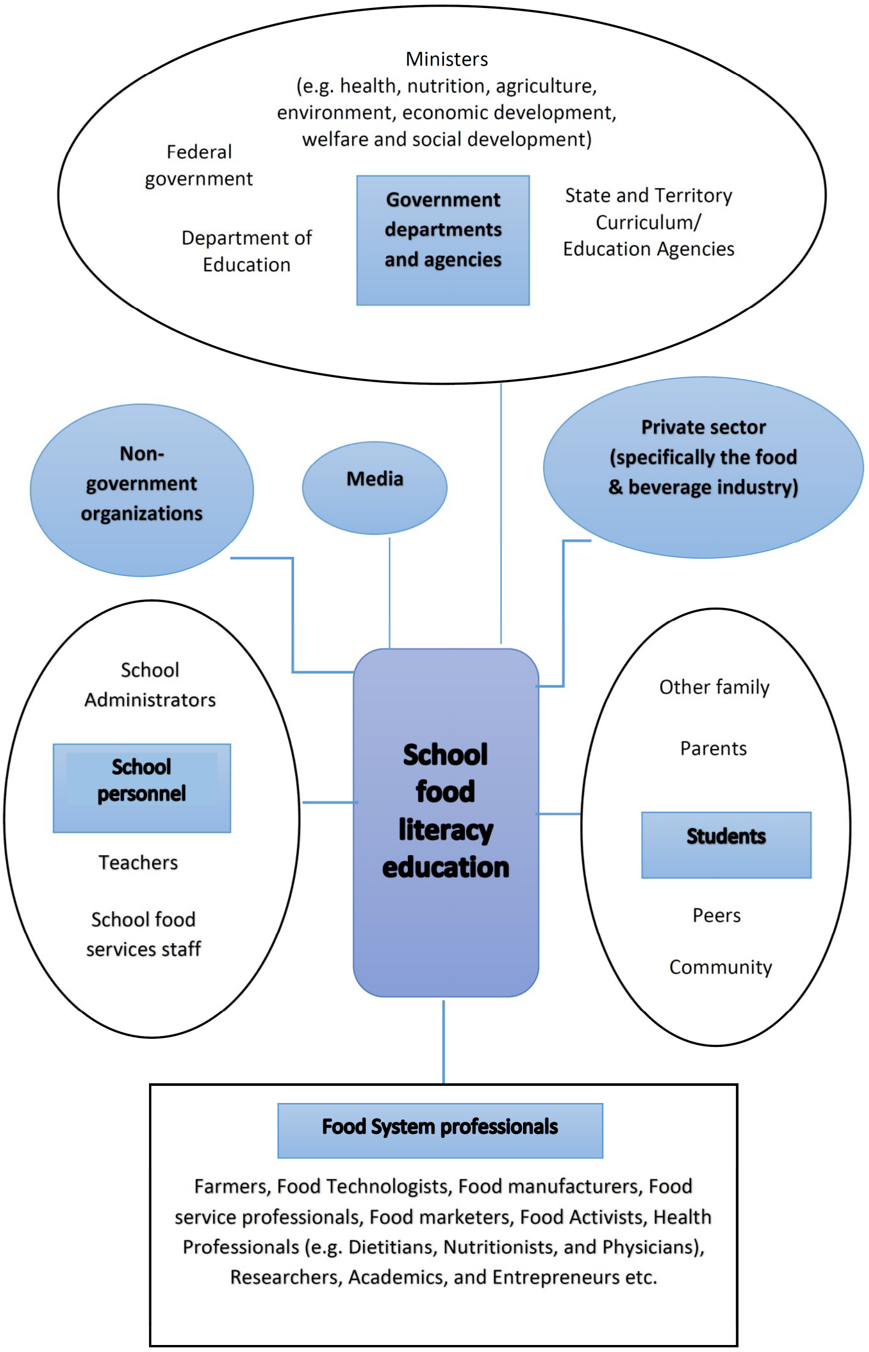

Figure S1: The model of stakeholder involvement in secondary school food literacy education

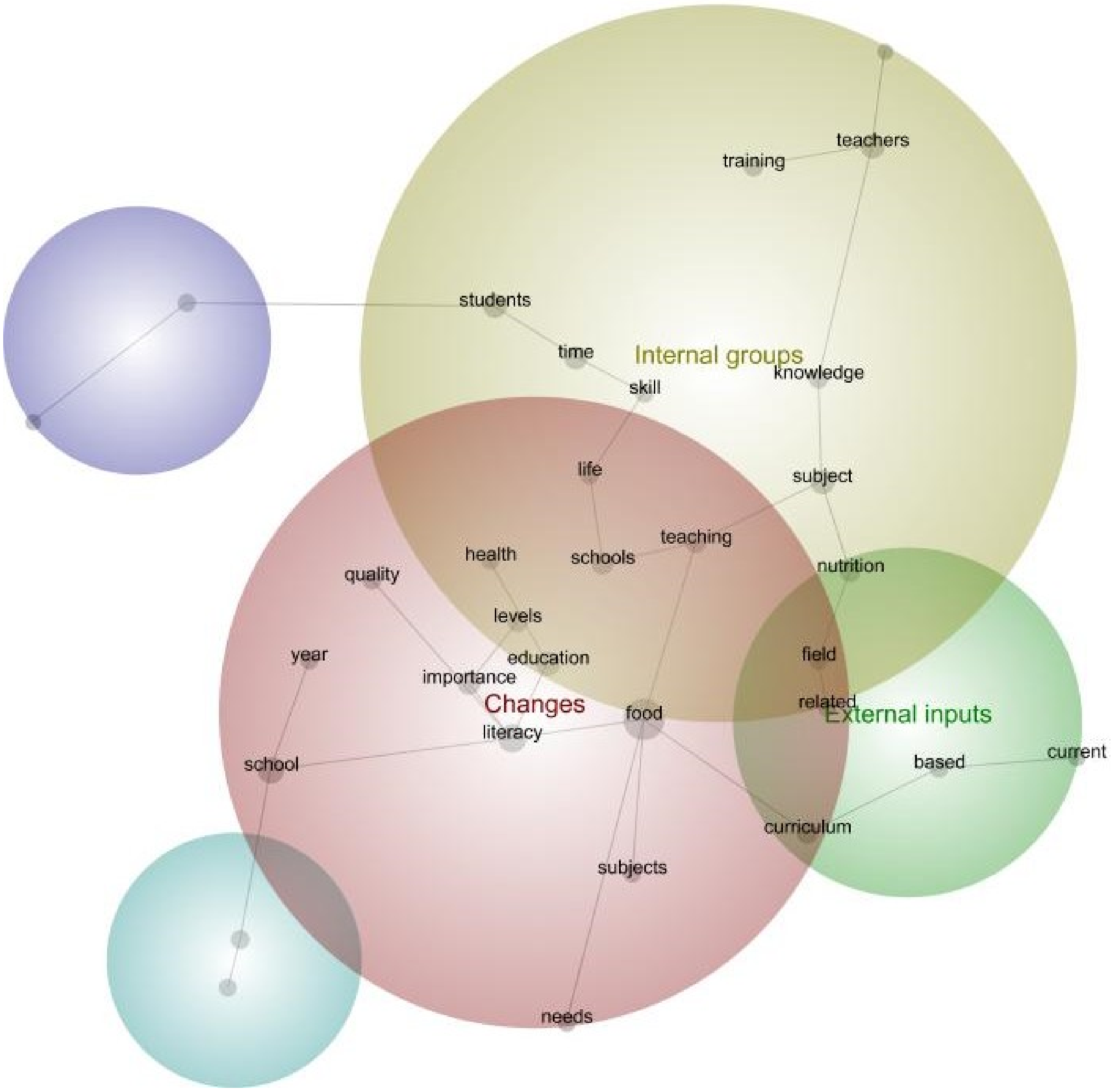

Figure S2: Leximancer concept map

Major themes generated by the Leximancer software and the associated concepts

CHANGES - Food, literacy, school, education, importance, schools, curriculum, levels, quality, year, health, subjects, needs

INTERNAL GROUPS - subject, teachers, students, time, nutrition, teaching, knowledge, training, skill, life, work

EXTERNAL INPUTS - based, field, related, current

Table S1. Percentages of respondents who agreed with the inclusion of different topics in the food literacy curriculum for senior secondary school students (years 11 and 12)

Statements	Agree (%)	Disagree (%)
Causes and prevention of food wastage	98	2
Planning and preparation of food in the home (resource management)	97	3
Safe food handling practices at home	97	3
Primary food production	95	5
Social influences, emotions and food consumption	95	5
The influence of food marketing on food choice	95	5
Consumers and food (passive and active food consumers)	94	6
The effects of cooking processes on food	94	6
World food problems	94	6
Design and adaptation of recipes to suit individuals' needs	92	8
Social media, mass media and food issues	92	8
The role of modelling in the establishment of healthy diets in children	92	8
Consumers' ethical concerns about food	92	8
Regulation of nutrient and health claims	92	8
The world food system and trade patterns	91	9
Current and emerging food trends	91	9
Nutritional quackery in the media and marketing	91	9
Food insecurity, access, and sovereignty	90	10
Biological reasons for differences in dietary requirements	89	11
Food governance and regulation	88	12
Application of evidence-based recommendations to everyday food practices	88	12
Water shortages and desertification	87	13
The environmental effects of the food system	87	13
The gastro-intestinal tract and digestive processes	86	14
The physiology of appetite and satiety	85	15
Climate change	84	16
The Australian Dietary Guidelines	83	17
World cuisines (e.g. Middle Eastern cuisines), Ethnic cuisines (e.g. Food consumed by people lived in Fertile crescent)	82	18
Indigenous food practices	81	19
Sensory food science principles	77	23
Food industry safety programs	76	24
Sensory evaluation of food products	76	24
Design and launch of new food products	74	26
Hunter gatherer and early agricultural food systems	66	34
Transferring domestic food skills to small scale commercial settings	66	34

Table S2. Respondents' agreement with seven statements related to students' activities and assessment-related tasks in food literacy education for senior secondary school students (years 11 and 12)

Statements	Agree (%)	Disagree (%)
The combination of theory and practical lessons helps students to develop food literacy knowledge and skills.	97	3
Practical classes or activities reinforce the food literacy concepts learnt during theory lessons.	96	4
Cooking should be an integral part of a food literacy subject.	94	6
Excursions (visits to food production, food processing, food serving and food distribution and retailing sites) should be included.	92	8
Guest lectures from experts in the food system are important for raising students' awareness of real-world food problems.	88	12
Short term internships and industry placements expose students to food- related careers.	81	19
Development of design briefs (new food product development plans) does not help to develop food literacy skills.	40	60

Table S3. Respondents' agreement on statements related to the barriers and challenges facing food literacy education for senior secondary school students

Statements	Agree (%)	Disagree (%)
Curriculum overload with other compulsory subjects (or competition with other subjects)	87	13
Exposure of students to conflicting food and nutrition-related information through different media (e.g. mass media)	85	15
Perceived low academic status of subject by school managements, parents and students	84	16
Lack of supportive school environment such as unhealthy school canteen food	78	22
Lack of support from school administration	77	23
Inadequate food guidance at home	76	24
Lack of awareness of food literacy concepts among teachers	73	26
Inadequate resources for teaching such as text books, kitchen facilities, money to buy ingredients for practicals	72	28
Lack of teacher training opportunities in relation to food literacy teaching	70	30
Lack of qualified teachers to properly teach the subject	65	35
Lack of relevance of food literacy education to future higher education pathways	58	42
Lack of relevance of food literacy education to future career pathways	57	43
Lack of resource sharing among teachers	50	50
Lack of students' interest on holistic food system approach in food literacy education	50	50
